# Infection Control Measures in Private Dental Clinics in Lebanon

**DOI:** 10.1155/2017/5057248

**Published:** 2017-05-31

**Authors:** Jihad Dagher, Charles Sfeir, Ahmad Abdallah, Zeina Majzoub

**Affiliations:** ^1^Department of Public Health Dentistry, Lebanese University, School of Dentistry, Hadath, Lebanon; ^2^School of Dental Medicine, University of Pittsburgh, Pittsburgh, PA, USA; ^3^Hammoud Hospital University Medical Center, Sidon, Lebanon; ^4^Department of Periodontics, Lebanese University, School of Dentistry, Hadath, Lebanon

## Abstract

**Purpose:**

Evaluate infection control knowledge, attitude, and practice in Lebanese private dental clinics.

**Materials and Methods:**

A survey including 46 questions related to routine safety procedures was sent to 1150 Lebanese dentists between July 1st and 2nd, 2015. The study sample was selected from the database of registered dentists based on a proportional random sampling ensuring equitable representation of the 5 geographic regions of Lebanon. A subset of 29 questions was used to generate an overall score of compliance (excellent, good, fair, and poor). Comparisons according to gender, type, region, and years of practice were performed.

**Results:**

417 dentists returned the completed questionnaires. 96% expressed concern about infection transmission, 90.6% were vaccinated against Hepatitis B, and 61.8% asked routinely about patients medical history. Only 43% used protective eyewear. Although most dentists (65%) used autoclaves, dry heat was still used. Significant correlations were found between gender and use of personal protective equipment. Less compliance was shown by clinicians with fewer years of experience. In the overall compliance questionnaire, the mean percentage of correct answers was roughly 54% with <5% of the practitioners scoring “excellent.”* Conclusions*. The study found inadequacy of compliance in private Lebanese dental clinics necessitating improved educational training and sustained monitoring by regulatory bodies.

## 1. Introduction

Infection continues to be one of the most critical issues in healthcare service worldwide. Infection prevention and control of cross-contamination are essential in providing a secure environment for patients and healthcare workers within healthcare settings in general and more specifically in dental practices. Transmission of infection during dental procedures may occur through direct contact with saliva, oral fluids, or blood, airborne droplets containing infective agents, or indirect contact via contaminated objects (e.g., instruments, equipment, or environmental surfaces) [1]. Exposure to blood-borne pathogens like human immunodeficiency viruses (HIV) and hepatitis B and C viruses (HBV and HCV) is a constant risk and therefore high standard precautions must be implemented and followed for all patients attending dental clinics regardless of their infection status [1]. Other infective agents such as Herpes viruses, varicella-zoster virus,* Mycobacterium* spp.,* Pseudomonas* spp.,* Legionella* spp., and multiresistant bacteria such as* Staphylococcus aureus* are scarcely documented but constitute additional risks of cross-contamination in dentistry [2].

Several healthcare bodies, agencies, and dental associations like the Centers for Disease Control and Prevention (CDC), Occupational Safety and Health Administration (OSHA), American Dental Association (ADA), the National Institute of Health and Clinical Excellence (NICE), and others have released guidelines to regulate infection control in the United States and other developed countries [1, 3–7]. In 2003, the CDC issued Guidelines for Infection Control in Dental Clinics. These recommendations include standard precautions which aim at ensuring a safe working environment and preventing potential transmission of occupational and nosocomial infections among dental personnel and their patients [1].

Compliance with effective infection control practices by dental healthcare providers may be affected by several factors such as knowledge and educational background [8, 9], costs and lack of incentives [10], sociodemographic and professional variables [11, 12], and availability of and access to required materials and equipment [13, 14]. Wide variations have been reported between developed and developing countries relative to infection prevention and control [14]. While several reports and systematic reviews have been published relative to compliance with infection control practices in dental healthcare of developed and developing countries [8, 10, 11, 13–34], surveys are limited and practically lacking in Lebanon.

Although Lebanon is considered to have low endemicity with low prevalence of HBV (1.74%) and HCV (0.21%) infections [35, 36], an increasing rate of other infectious diseases such as tuberculosis (TB) and HIV/AIDS [37] is being observed in the Middle East and North African regions and is likely to affect Lebanon [38] due to the influx of nonnationals and refugees.

The objectives of the present study are to investigate knowledge, attitude, and practices relative to infection control measures in private dental clinics in Lebanon.

## 2. Materials and Methods

### 2.1. Sample Selection

The study was conducted using a survey questionnaire including questions on various aspects of routine infection control measures in dental clinics. The sample size to be included in the survey was calculated using the sampling formula:

(1) $$\begin{array}{c} Sample=\frac{Nz^{2}pq}{d^{2}\left(N-1\right)+z^{2}pq}, \end{array}$$

where

- *N* is the total population of dentists registered in the Lebanese Dental Associations;
- *p *is estimated proportion of dentists who are assumed to adhere to infection control guidelines (50% as no other data is available);
- *q* = 1 − *p*;
- *z *is the number of standard deviation away from the sample proportion (*z* = 1.96 for a 95% confidence interval);
- *d *is half of the width of the confidence interval of the sample proportion* p* (*d *= 5% and confidence interval = 50 ± 5%, which means 45% ≤* p* ≤ 55%).

The sample size considered statistically representative for the objectives of the study was 359 according to the abovementioned formula. This figure represents 7.13% of the total number of Lebanese dentists. The sampling frame was a database of all dentists registered in the Lebanese Dental Associations and was provided by the Associations boards. Dentists were selected based on a proportional stratified random sampling to ensure equitable representation of all 5 geographic regions of Lebanon. Participating dentists were drawn from various sociodemographic and professional contexts regardless of age, gender, type, or years of practice. When a dentist declined to participate in the study, he/she was substituted by the following dentist on the list.

### 2.2. Questionnaire

Assuming a response rate of approximately 30%, questionnaires were sent via email to a sample of 1150 dentists between July 1st and 2nd, 2015. Participants who did not respond to the first mailing were reminded 3 weeks later through an emailed memo. All questionnaires were forwarded with a cover letter explaining the goals of the study. Given the lack of a standardized validated questionnaire to assess knowledge, attitude, and practices of infection control measures by dentists, a pilot survey was conducted on a random sample of 50 dentists working in private clinics to ensure practicability, relevance, and proper interpretation of the questions. The responses from the pilot test were analyzed and the questionnaire was modified according to the feedback obtained. The finalized questionnaire consisted of 46 questions with mainly categorical answers (apart from the number of years in practice) covering 9 issues selected according to the CDC guidelines:

- *Section  1*. It covers general information with 4 questions related to gender and type of practice (i.e., general versus specialty practice, years of experience, and main region of practice).
- *Section  2*. It covers infection control knowledge and awareness with 11 questions related to the sources of infection control knowledge, infectious diseases and transmission of infection, record of patients medical history, and dentist and staff HBV immunization.
- *Section  3. *It covers hand hygiene (with 1 question related to the frequency of hand washing) and personal protective equipment with 8 questions covering the use of gloves, protective eyewear, mask, head cover, disposable items, and gowns.
- *Section  4. *It covers control of aerosol with 3 questions about use of rubber dam, high volume evacuator, and preoperative mouth rinses.
- *Section  5. *It is represented by 5 questions about occupational accidents (sharp injuries record and treatment protocol), disposal of sharp instruments, and medical waste management.
- *Section  6. *It includes 8 questions addressing cleaning, disinfection, and sterilization of instruments, burs, and handpieces.
- *Section  7. *It covers surface barriers and surface disinfection with 4 questions (computer keyboard, curing light source, and dental unit surfaces).
- *Section  8. *It includes 1 question related to impression disinfection.
- *Section  9. *It includes 1 question related to time interval between patients on the same dental chair.

### 2.3. Statistical Analysis

Questionnaires with less than 90% of completed answers were excluded. The answers were recorded and processed using the Statistic Package for the Social Sciences (IBM SPSS for Windows, Version 20.0; IBM Corp., Armonk, NY, USA). Descriptive statistics and frequency distributions were generated for all variables. Chi-square test was used in bivariate analysis to assess differences in infection control knowledge, attitude, and practice according to gender, qualification (general practitioner versus specialist), geographic location of practice, and years of experience. Overall compliance with infection control measures was assessed separately using a subset of 29 pertinent questions (see Appendix) and the percentage of questions answered correctly was calculated for all surveyed clinicians. Responses were considered correct if the answer was yes to questions (1)–(5) and (18)–(20), if the frequency of adherence to a specific practice was confirmed as being “always” (questions (6)–(17), (21), and (23)–(29)), and if instruments were reported to be decontaminated prior to washing (question (22)). The scores were judged excellent if more than 85% of the questions were answered correctly, good if the percentage of correct questions ranged between 66 and 85%, fair if 50% to 65% of the answers were exact, and poor if less than 50% were correct. One-way ANOVA followed by Bonferroni Post Hoc tests were used to compare mean overall compliance by gender, type of practice, region of practice, and years of experience. Chi-Square was applied to compare categories of compliance (excellent, good, fair, and poor) across the 4 sociodemographic and professional variables. Statistical significance was set at *p* < 0.05. This research was conducted in full accordance with the World Medical Association Declaration of Helsinki and all responses obtained from participants were blinded and kept confidential.

## 3. Results

Out of the 1150 dentists included in the initial sample, 420 respondents returned the completed data forms yielding a response rate of 36.3%. All the filled-out questionnaires were received between August 18th and November 3rd, 2015.  Table 1 summarizes the distribution of dentists according to gender and type, years, and region of practice.

Table 2 illustrates knowledge, attitude, and behavior of the participating Lebanese dentists concerning infectious diseases. The main source of knowledge related to infection control was reported to be graduate dental school courses (89.5%). Nearly all respondents (96%) expressed concern about risks of infection transmitted in dental practices. The mode of transmission through splatter was recognized by 92.9% of the participants while the percutaneous route was acknowledged by only 72.1%. More than 40% of the surveyed persons (43.8%) considered AIDS to be the most worrisome potential threat in dental practice. The rate of vaccination against Hepatitis B was very high among dentists (90.2%) but only 72.6% indicated having had a subsequent booster dose of vaccine. The corresponding percentages among the oral health care staff (dental assistants) were lower (34% and 28.6% resp.). About 62% of the participants reported reviewing the medical history of their patients prior to initiating treatment. Nearly half of the respondents (51.9%) believed that dentists have the right to refuse dental care of patients with infectious diseases.

**Figure 1:**
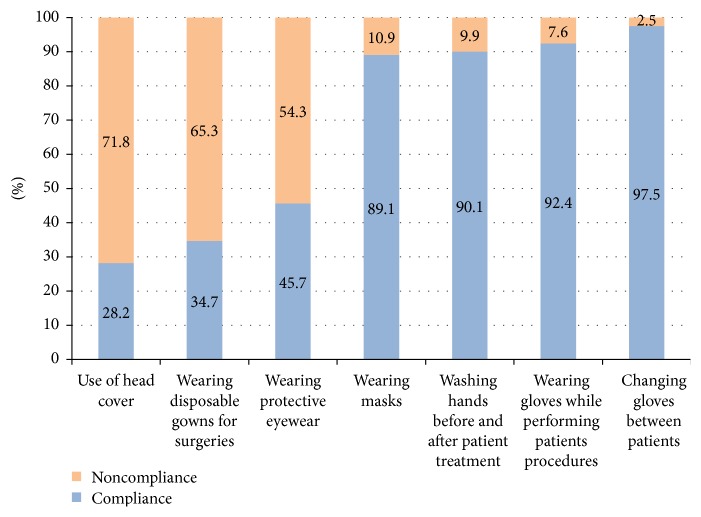
Graph summarizing hand hygiene practices and use of personal protective equipment among the surveyed Lebanese dentists (2015).

Figure 1 summarizes the compliance of the surveyed clinicians with hand hygiene practices and use of personal protective equipment. While gloves and face masks tended to be routinely used and changed by most practitioners, head covering, disposable gowns for surgery, and protective eyewear were less widely implemented (28.2%, 34.7%, and 45.7% resp.).

**Figure 2:**
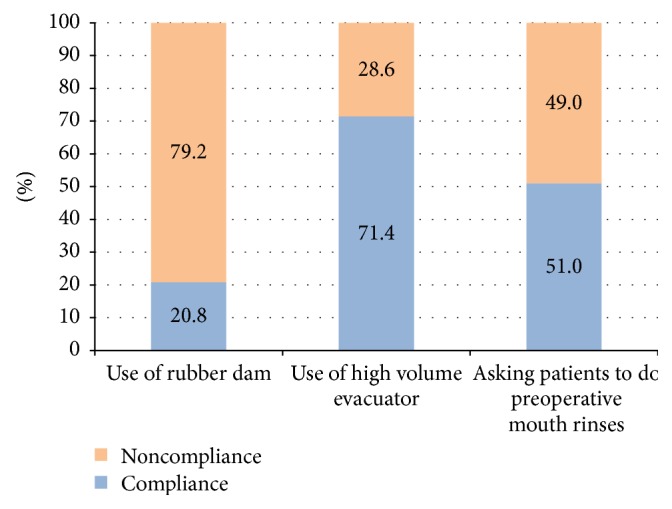
Graph showing compliance with aerosol control in Lebanese private dental clinics (2015).

When control of aerosol was assessed (Figure 2), a low rate of rubber dam use was reported (20.8%). Nearly half of the dentists (51.0%) asked their patients to rinse prior to dental procedures and 71.4% used high volume evacuators.

**Figure 3:**
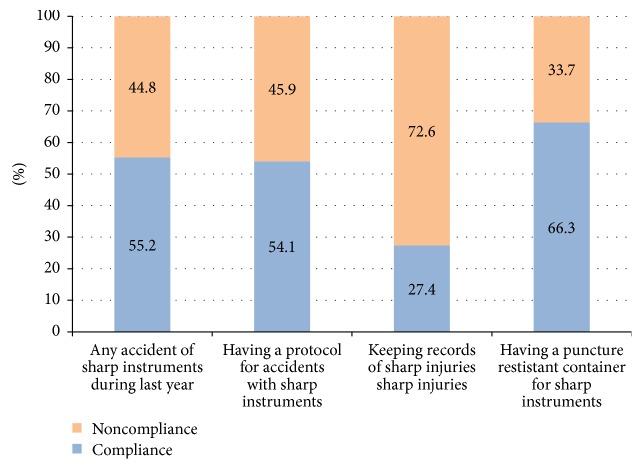
Graph summarizing the occurrences of sharp injuries and prophylactic measures adopted in the surveyed Lebanese private clinics (2015).

Occupational hazards occurrences as well as prophylactic measures taken in case of sharp injuries are summarized in Figure 3. Sharp incidents were experienced by more than half of the practitioners and/or their dental assistants (55.2%). Only 27.4% kept records of such accidents and 54.1% had an appropriate protocol to manage them. Special puncture resistant containers for sharps disposal were available in 66.3% of the surveyed clinics. Only 19% of the respondents disposed of medical waste through specialized companies.

Table 3 shows that although the autoclave seems to be the preferred means of sterilization (65%), dry heat sterilizers (35.0%) continue to be used among the respondents. Approximately two-thirds of the participants reported heat-sterilizing endodontic files (60.3%) and burs (65.3%). Instruments were immersed in decontaminant solutions mainly prior to washing (65%) and were for the most part manually scrubbed (79.0%). Nearly half (55.1%) of the surveyed dentists applied impermeable barriers on clinical contact surfaces. Routine wiping of working surfaces with surface disinfectant was reported by 81% of the respondents. Approximately half of the practitioners (52.4%) allowed 5- to 15-minute intervals between patients. Most of these practitioners leaving relatively long intermissions between consecutive patients practiced routine wiping of clinical contact surface areas (91%). Only 38.4% of the participants reported using chemical disinfectants routinely to treat impressions before sending them to the laboratory.

Potential correlations between sociodemographic and professional variables (gender, years of experience, location, and qualification) and HBV vaccination, hand hygiene, and use of personal protective equipment were evaluated using the Chi-square test (Table 4). Female dentists reported a higher rate of wearing protective eyewear (*p* = 0.006) and disposable gowns for surgical procedures (*p* = 0.037) than their male colleagues. Clinicians with fewer years of service were less compliant than those with more than 20 years of experience relative to hands washing practices (*p* = 0.011), wearing masks (*p* = 0.024), and protective eyewear (*p* = 0.041). Significantly more dentists working in Beirut and Mount Lebanon adhered to HBV vaccination (*p* = 0.010), washing hands (*p* = 0.003), and wearing gloves (*p* = 0.019) and face masks (*p* = 0.039). The level of education of the respondents (general practitioners versus specialists) did not significantly affect any of the variables evaluated. The use of rubber dam was significantly associated with gender and practice location with female respondents and dentists practicing in Beirut and Mount Lebanon reporting a higher rate of use of rubber dam (*p* = 0.004 and 0.009, resp.).

Overall, compliance with the infection control measures listed in the Appendix was poor in the surveyed sample with a percentage of correct answers ranging between 50% and 58% (Table 5). Less than 5% of the surveyed dentists were considered to have excellent levels of compliance (more than 85% of correct answers) while approximately 27% and 35% of the respondents had fair or poor compliance scores, respectively. The level of compliance was not significantly affected by the sociodemographic and professional variables.

## 4. Discussion

This study attempted to assess knowledge, attitude, and practice of infection control in the private dental sector in Lebanon. Although the present investigation did not evaluate the full range of issues related to infection control, it has, similarly to most published studies, focused on most common categories of items directed towards compliance with specific procedures, such as hand hygiene, use of personal protective equipment, occupational hazards and vaccination, medical records, decontamination, and sterilization. It should also be noted that this survey relies on self-reported information and might therefore represent an overestimation of correct infection control knowledge and practice among Lebanese dentists.

The response rate to the distributed questionnaire in the present study was very low (36.3%) in contrast with Jordanian (91.66%) [32] and Saudi (98.5%) [34] figures in comparable surveys. This low response rate emphasizes the lack of interest/awareness or the low level of importance that Lebanese private dentists associate with the issue of infection control.

Another general finding is that Lebanese practitioners widely apply certain basic infection control measures such as hand hygiene (90.1%), use of gloves (92.4%) and masks (89.1%), and vaccination against HBV (90.6%) but poorly address other significant issues such as occupational hazards, medical history records, disinfection of impressions, disposal of hazardous dental waste, and sterilization of handpieces, endodontic instruments, and burs. This resulted in fair to poor levels of overall compliance in nearly two-thirds of the surveyed dentists. These findings are comparable with the general conclusions of studies conducted in private dental clinics in other Middle Eastern Arabic countries such as Jordan [32, 33] and Saudi Arabia [34]. This can be attributed to adequate basic infection control programs in dental schools but subsequent lack of constant reinforcement through continuing education courses and regularly updated recommendations circulated through dental schools, dental associations, and governmental agencies.

Although legal decrees regulating healthcare waste management have been issued in 2004 by the Ministry of Environment [39], its implementation has not been proficiently extended to dental care facilities. It is interesting to note that 66.3% of the respondents had dedicated puncture resistant containers for sharp disposal, but only 19% disposed of medical waste through specialized companies. The figures related to sharp segregation in appropriate containers are better than those reported by Daou et al. [40] (28%) in Lebanese dental clinics. These discrepancies could be attributed to the larger sample and different region distribution in the present study. In fact North Lebanon—where the use of dedicated containers for sharp disposal is less common (48.0%) than Mount Lebanon (70.6%)—is less represented in the present sample while the percentage of surveyed clinics from Mount Lebanon is higher. In addition, the present findings being 4 years more recent than Daou's results is likely to reflect some improvements in sharp collection. This positive trend is still however associated with disposal of approximately 47% of the sharp containers generated by the surveyed sample in municipal waste.

The majority of surveyed dentists in the present sample were aware of infectious risks associated with dental procedures (96%) and had high HBV vaccination rates (90.6%) comparable to those observed in developed countries. This high compliance is likely due to perceived risks of contracting HBV infection, understanding of the merits of vaccination, and availability of the vaccine. However, this high vaccination rate was coupled with severe deficiencies related to booster immunization, dental staff HBV vaccination, sharp injury records, postexposure prophylaxis protocols, and irregular use of protective eyewear and clothing. The present study did not conduct any serologic testing to assess if self-reported vaccination among the surveyed dentists reflected their immunization and antibody status. It is likely that at least some of the participants are not fully immunized and protected since only 72.6% reported having had their booster shots. Only 34% of the dental staff received HBV vaccination. This is consistent with other surveys where dental auxiliaries were less likely to be vaccinated than the dentists themselves [41–43] although the annual number of sharp injuries outside hospital facilities and resulting HBV infections have been reported to be higher among dental assistants than among dentists [44]. The wide application of hand scrubbing of instruments (79%) is likely to be associated with a higher prevalence of accidental percutaneous injuries among the mostly underqualified Lebanese dental assistants. Effective educational strategies relative to prevention and postexposure protocols should be implemented among Lebanese dentists and their auxiliary dental staff. This must be coupled with constant monitoring of occupational injuries and related safety practices and provision of nationwide vaccination programs through dedicated occupational health services. The requirement of healthcare certificates confirming immunization status should be instigated among dental practitioners and staff as a prerequisite for practice or license renewal.

Most surveyed dentists in the present study considered HIV to be the most worrisome infectious disease (43.8%) in comparison to 35% for HBV although the risk of acquiring HBV has been reported to be significantly greater than that of acquiring HIV among nonimmune dental anesthesiologists [45] and the mortality risk of HBV infection is greater than HIV [46]. Similar trends of assigning higher levels of occupational risk to HIV infection were reported in a study assessing the willingness of Palestinian dentists to treat patients with blood-borne diseases [47] where 68% of the respondents declined appointment requests from patients with HIV versus 32% for patients with HBV.

Although it is considered unethical to refuse dental care of patients with blood-borne infections, nearly 52% of the Lebanese participants believed that dentists have the right to refuse treating patients with such pathologies. This prejudice towards infected individuals is a common finding in Middle Eastern countries [48–51]. Such attitude is likely to be associated with the belief that dentists do not have the ethical responsibility to provide care for infected subjects. Patients facing such reluctance may tend to hide their infection status from their dentists with negative implications on cross-infection. Emphasis on ethics training at the undergraduate and postgraduate levels and improved knowledge of infectious diseases should be implemented to ensure proper management of patients with or at high risk of blood-borne infections.

The overall level of compliance with the infection control measures listed in the Appendix was not significantly affected by any of the sociodemographic or professional variables. However adherence to some specific infection control practices was significantly different between genders such as use of protective eyewear and disposable gowns with women showing better compliance than their male counterparts. This finding replicates the results of previously published surveys [12, 52, 53] where women are more likely to make frequent use of certain personal protective equipment without however showing greater general compliance levels.

When the remaining sociodemographic and professional variables were considered in the present study, dentists in practice for more than 20 years demonstrated more compliance with hands hygiene and use of personal protective equipment (eyewear and mask) than practitioners with fewer years of professional experience. This finding is not universal in surveys involving the private dental sector in different countries [34, 54, 55]. Although older dentists with more experience might have had little information related to infection control during their graduate studies, they could have, in some cases, acquired considerable knowledge during their longer professional experience. The lack of significant differences between specialists and general practitioners is in line with the findings of other investigators [56]. It should be noted however that the present study did not attempt to identify the type of specialty (oral surgery, orthodontics, periodontics, etc.) practiced in the surveyed sample. It is possible that oral surgery specialized dental practices implement stricter infection control measures than other specialties or general dentistry-practicing clinics [8]. The highest rates of HBV vaccination, use of masks, and hands hygiene practices were found among dental practitioners from Beirut and Mount Lebanon. More dentists practicing in these same two areas were also reported to have eliminated amalgam use in their clinics [40]. This trend in the capital and Mount Lebanon compared to the remaining Lebanese regions can be anecdotally attributed to differences in financial constraints and patients' perception since these two areas are more advantaged economically and benefit from greater demand for high quality dental care.

## 5. Conclusions

In conclusion, the results of the present study highlight the inappropriate knowledge, attitude, and practice relative to infection control in the Lebanese private dental sector. While implementation of selective standard precautions is highly practiced, the overall compliance remains poor. Further studies should be designed to identify the reasons behind such poor compliance and barriers for the generalized implementation of CDC infection control guidelines in Lebanon. Some tangible steps should be considered by the Ministry of Health in collaboration with the Lebanese Dental Associations and regulated under a formalized legal legislation: (1) development of strategies targeted towards raising awareness of the importance of infection control among Lebanese dental students and dentists; (2) increasing related continuing education requirements and mandatory courses/workshops; (3) development and distribution of infection control manuals that incorporate updated guidelines and recommendations for dental practices; (4) continuous monitoring of private dental clinics to supervise adherence to standard CDC guidelines; and (5) instigating certain practices—such as vaccination and confirmation of the immunization status of dentists and dental auxiliaries—as a prerequisite for practice or license renewal. Training of dental assistants in infection control practices should be implemented through mandatory continuing education courses initially and subsequently through formal educational programs in dental assisting.

## Figures and Tables

**Table 1:** Sociodemographic and professional distribution of the surveyed dentists in private dental clinics in Lebanon (2015). The difference in the total number of respondents for each variable is due to missing data in the filled-out questionnaire forms.

Characteristic	*N*	%
*Gender*		
Female	173	41.5
Male	244	58.5
*Type of practice*		
General practitioner	243	58.7
Specialist	171	41.3
*Region of practice*		
Beirut	103	24.9
Beqaa	39	9.4
Mount Lebanon	170	41.2
South Lebanon and Nabatieh	50	12.1
North Lebanon	51	12.3
*Years of experience*		
≤5	83	20.0
6–10	71	17.1
11–15	76	18.3
16–20	82	19.7
>20	104	25.0

**Table 2:** Knowledge, attitude, and behavior of dentists relative to infectious diseases in private dental clinics in Lebanon (2015).

Questions related to infectious diseases	Number of respondents	%
*Source of knowledge about infection control*		
Dental school courses	376	89.5
Scientific meetings	134	31.9
Postgraduate courses	99	23.6
Dental journals	86	20.5
Other	41	9.8
*Knowledge about transmission of infectious diseases*		
Infection can be transmitted in dental practice	403	96.0
Infection can be transmitted through splatter/splash	390	92.9
Infection can be transmitted through percutaneous route	303	72.1
*Asking about medical history*	260	61.8
*Staff and dentists HBV vaccination status*		
The dentist is vaccinated against Hepatitis B	356	90.6
The dentist took the booster shot against Hepatitis B	273	72.6
The dental staff is vaccinated against Hepatitis B	127	34.0
The dental staff took the booster shot against Hepatitis B	103	28.6
*Dentists have the right to refuse care of infected patients*	218	51.9
*Vaccination is the most predictable way for prevention of Hepatitis B*	379	90.2

**Table 3:** Cleaning, disinfection, and sterilization practices among private Lebanese dental clinics (2015).

Questions related to cleaning, disinfection, and sterilization	Number of respondents	%
*Method of cleaning used instruments*		
Manual washing	332	79.0
Ultrasonic cleaner	71	16.9
Washer disinfector	35	8.3
*Timing of immersion of the used instruments in decontaminant solution*		
Before washing	225	65.0
After washing	121	35.0
*Kind of sterilizer used*		
Dry heat	133	31.7
Autoclave	273	65.0
*Use of heat sterilization for handpieces*	98	27.7
*Use of heat sterilization for burs*	226	65.3
*Use of heat sterilization for endodontic files*	210	60.3
*Use of wrapping bags for instruments sterilization*	250	70.4
*Covering computer keyboards in clinical areas*	104	29.8
*Use of surface barriers for dental unit surfaces*	193	55.1
*Use of any surface disinfectant for routine wiping*	278	81.0
*Time interval between patients on the same dental chair*		
<5 minutes	130	36.0
5–15 minutes	189	52.4
>15 minutes	42	11.6
*Disinfect impressions before sending to the laboratory*	132	38.4

**Table 4:** Adherence to some selected infection control practices by Lebanese private dentists according to sociodemographic variables and work experience (2015). ^*∗*^Statistically significant differences within each sociodemographic and professional group using the Chi-square test.

	Hand washing	Gloves	Masks	Protective eyewear	Disposable gowns for surgery	HBV vaccination	Use of puncture resistant sharp-container	Instrument immersion before cleaning
*Gender*								
Female	88.8%	91.5%	86.6%	52.2%	37.2%	90.1%	68.3%	79.1%
Male	91.3%	93.4%	91.2%	37.0%	33.3%	90.8%	64.6%	56.2%
*p* value	0.414	0.204	0.182	0.006^*∗*^	0.037^*∗*^	0.446	0.446	0.000^*∗*^
*Type of practice*								
General practitioner	89.0%	91.8%	89.1%	45.9%	35.9%	88.6%	67.1%	58.9%
Specialist	92.5%	93.7%	90.0%	46.5%	33.6%	93.2%	66.2%	73.6%
* p* value	0.245	0.632	0.145	0.499	0.546	0.280	0.860	0.005^*∗*^
*Region of practice*								
Beirut	90.9%	93.3%	96.6%	54.0%	40.0%	96.6%	69.3%	69.9%
Beqaa	77.1%	94.6%	86.5%	37.8%	32.4%	77.8%	63.9%	54.5%
Mount Lebanon	95.8%	96.4%	91.5%	44.8%	36.8%	89.2%	70.6%	64.4%
South Lebanon and Nabatieh	85.7%	87.5%	81.6%	40.8%	30.4%	95.8%	68.8%	66.7%
North Lebanon	84.3%	82.4%	80.0%	48.0%	27.7%	90.2%	48.0%	64.6%
*p value*	0.003^*∗*^	0.019^*∗*^	0.039^*∗*^	0.452	0.210	0.010^*∗*^	0.052	0.660
*Years of experience*								
≤5	88.2%	93.6%	93.6%	49.4%	33.3%	93.5%	78.2%	86.6%
6–10	80.0%	91.4%	81.4%	35.7%	30.3%	92.9%	58.0%	64.6%
11–15	95.9%	90.5%	87.8%	31.1%	33.3%	91.9%	67.6%	62.0%
16–20	94.9%	94.9%	88.5%	47.4%	38.9%	88.6%	64.9%	61.8%
>20	90.3%	91.2%	92.3%	60.4%	35.4%	86.8%	61.8%	52.7%
*p* value	0.011^*∗*^	0.791	0.024^*∗*^	0.041^*∗*^	0.296	0.490	0.092	0.001^*∗*^

**Table 5:** Levels of compliance with infection control practices in private dental clinics in Lebanon (2015) according to sociodemographic variables and work experience. The level of compliance is expressed as excellent, good, fair, or poor according to the percentage of correct answers to the questions listed in the Appendix. The difference in the total number of respondents for each variable is due to missing data in the filled-out questionnaire forms.

Factor	Excellent compliance *(86–100% of correct answers)*	Good compliance *(66–85% of correct answers)*	Fair compliance *(50–65% of correct answers)*	Poor compliance *(<50% of correct answers)*	Percentage of correct answers *(mean ± SD)*
*Gender*					
Female (*n* = 173)	2.3%	40.5%	23.1%	34.1%	54.93 ± 21.99
Male (*n* = 244)	6.6%	27.0%	29.9%	36.5%	53.67 ± 21.99
* Total (n = 417)*	*4.8%*	*32.6%*	*27.1%*	*35.5%*	*54.20 ± 21.97*
*Type of practice*					
General practitioner (*n* = 243)	6.2%	31.7%	28.0%	34.2%	54.70 ± 22.54
Specialist (*n* = 171)	2.9%	34.5%	26.3%	36.3%	54.06 ± 20.72
* Total (n = 414)*	*4.8%*	*32.9%*	*27.3%*	*35.0%*	*54.44 ± 21.78*
*Region of practice*					
Beirut (*n* = 103)	7.8%	28.2%	26.2%	37.9%	52.90 ± 24.11
Beqaa (*n* = 39)	2.6%	33.3%	17.9%	46.2%	50.40 ± 24.52
Mount Lebanon (*n* = 170)	3.5%	38.2%	31.2%	27.1%	58.36 ± 18.34
South Lebanon/Nabatieh (*n* = 50)	6.0%	26.0%	24.0%	44.0%	50.90 ± 23.57
North Lebanon (*n* = 51)	3.9%	29.4%	27.5%	39.2%	51.86 ± 21.24
* Total (n = 413)*	*4.8%*	*32.7%*	*27.4%*	*35.1%*	*54.54 ± 21.66*
*Years of experience*					
≤5 (*n* = 83)	6.0%	33.7%	30.1%	30.1%	57.00 ± 20.74
5–10 (*n* = 71)	7.0%	31.0%	25.4%	36.6%	53.42 ± 22.75
11*–*15 (*n* = 76)	3.9%	36.8%	31.6%	27.6%	57.08 ± 19.12
16*–*20 (*n* = 82)	2.4%	39.0%	23.2%	35.4%	55.26 ± 20.87
>20 (*n* = 104)	3.8%	25.0%	26.9%	44.2%	49.57 ± 24.03
* Total (n = 416)*	*4.6%*	*32.7%*	*27.4%*	*35.3%*	*54.20 ± 21.80*

## Appendix

Subset of 29 questions related to overall compliance with infection control measures. All answers were categorical and included “yes” or “no” for questions (1)–(5) and (18)–(20), “before” or “after” for question (22), and the 4-point scale “always,” “occasionally,” “rarely,” and “never” for the remaining questions. Answers were scored as correct for the “yes,” “before,” and “always” replies.

*Practice Compliance Questionnaire*

1. Do you routinely ask your patients about their medical history?
2. Are you vaccinated against Hepatitis B?
3. Did you take your booster shot?
4. Is your dental staff (assistants) vaccinated against Hepatitis B?
5. Did your staff take their booster shot?
6. How often do you wash hands before and after patient treatment?
7. How often do you wear gloves while performing dental procedures?
8. How often do you change gloves between patients?
9. How often do you use sterile surgical gloves for surgery?
10. How often do you wear protective eyewear?
11. How often do you wear mask?
12. How often do you change masks between patients?
13. How often do you use head covering?
14. How often do you wear disposable gowns for surgery?
15. How often do you use rubber dam?
16. How often do you use high volume evacuator?
17. How often do you ask your patient to do preoperative mouthrinses?
18. Do you have an appropriate protocol for emergency treatment of needle stick or other sharp accidents?
19. Do you keep detailed records of these accidents?
20. Do you use puncture resistant container for sharp instruments?
21. How often do you do you immerse the used instruments in decontaminant solutions?
22. When do you immerse the used instruments in decontaminant solutions?
23. How often do you use heat sterilization for handpieces?
24. How often do you use heat sterilization for burs?
25. How often do you use heat sterilization for endodontic files?
26. How often do you use wrapping bags for instruments sterilization?
27. How often do you use surface barriers for dental unit surfaces?
28. How often do you use surface disinfectants for routine wiping?
29. How often do you chemically disinfect impressions before sending to the laboratory?

## References

[1] Kohn W. G., Collins A. S., Cleveland J. L., Harte J. A., Eklund K. J., Malvitz D. M. (2003). CDC centers for disease control and prevention guidelines for infection control in dental health care settings. *MMWR Recommendations and Reports*.

[2] Laheij A. M. G. A., Kistler J. O., Belibasakis G. N., Välimaa H., de Soet J. J. (2012). Healthcare-associated viral and bacterial infections in dentistry. *Journal of Oral Microbiology*.

[3] OSHA Occupational Safety and Health Administration (2010). OSHA instruction bloodborne pathogens exposure control plan and guidance on post-exposure evaluations for federal osha personnel. *Directive no.*.

[4] American Dental Association (2016). *ADA Guidelines for Infection Control*.

[5] National Institute for Health and Care Excellence (NICE) (2016). *Evidence-Based Information for Health, Public Health and Social Care Professionals*.

[6] Lemass H., McDonnell N., O'Connor N., Rochford S. (2014). *Infection Prevention and Control for Primary Care in Ireland: A Guide for General Practice*.

[7] Australian guidelines for the prevention and control of infection in healthcare. NHMRC guidelines. CD33, 2010

[8] Tada A., Watanabe M., Senpuku H. (2014). Factors influencing compliance with infection control practice in Japanese dentists. *The International Journal of Occupational and Environmental Medicine*.

[9] Ebrahimi M., Ajami B. M., Rezaeian A. (2012). Longer years of practice and higher education levels promote infection control in Iranian dental practitioners. *Iranian Red Crescent Medical Journal*.

[10] Al Shatrat S. M., Shuman D., Darby M. L., Jeng H. A. (2013). Jordanian dentists' knowledge and implementation of eco-friendly dental office strategies. *International Dental Journal*.

[11] Cheng H. C., Su C. Y., Huang C. F., Chuang C. Y. (2012). Changes in compliance with recommended infection control practices and affecting factors among dentists in Taiwan. *Journal of Dental Education*.

[12] Breda-Albuquerque F., de Farias A. B., do Prado M. G., Orestes-Cardoso S. (2008). Influence of clinicians' socio-demographic, professional and educational variables on their compliance with preventive measures against hepatitis B and C. *Oral Health and Preventive Dentistry*.

[13] Puttaiah R., Miller K., Bedi R. (2011). Comparison of knowledge, attitudes and practice of dental safety from eight countries at the turn of the Century. *Journal of Contemporary Dental Practice*.

[14] Oosthuysen J., Potgieter E., Fossey A. (2014). Compliance with infection prevention and control in oral health-care facilities: a global perspective. *International Dental Journal*.

[15] Moradi Khanghahi B., Jamali Z., Pournaghi Azar F., Naghavi Behzad M., Azami-Aghdash S. (2013). Knowledge, Attitude, practice, and status of infection control among Iranian dentists and dental students: a systematic review. *Journal of Dental Research, Dental Clinics, Dental Prospects*.

[16] Smith A. J., Wilson S. L., Read S. (2014). Patients' perception of infection prevention in dental practice. *American Journal of Infection Control*.

[17] Oosthuysen J., Potgieter E., Blignaut E. (2010). Compliance with infection control recommendations in South African dental practices: a review of studies published between 1990 and 2007. *International Dental Journal*.

[18] Cleveland J. L., Bonito A. J., Corley T. J. (2012). Advancing infection control in dental care settings: factors associated with dentists' implementation of guidelines from the Centers for Disease Control and Prevention. *Journal of the American Dental Association*.

[19] Budnyak M. A., Gurevich K. G., Fabrikant K., Miller K., Puttaiah R. (2012). Dental infection control and occupational safety in the Russian federation. *Journal of Contemporary Dental Practice*.

[20] Su J., Deng X.-H., Sun Z. (2012). A 10-year survey of compliance with recommended procedures for infection control by dentists in Beijing. *International Dental Journal*.

[21] Uti O. G., Agbelusi G. A., Jeboda S. O., Ogunbodede E. (2009). Infection control knowledge and practices related to HIV among Nigerian dentists. *Journal of Infection in Developing Countries*.

[22] Halboub E. S., Al-Maweri S. A., Al-Jamaei A. A., Tarakji B., Al-Soneidar W. A. (2015). Knowledge, attitudes, and practice of infection control among dental students at Sana'a University, Yemen. *Journal of International Oral Health*.

[23] Alduais A. M., Mogali S. G. (2015). Assessment of infection control in dental clinics at Ibb City, Republic of Yemen: dentists perspective. *Science Journal of Public Health*.

[24] Ahmad I. A., Rehan E. A., Pani S. C. (2013). Compliance of Saudi dental students with infection control guidelines. *International Dental Journal*.

[25] Barghout N., Al Habashneh R., Ryalat S. T., Asa'ad F. A., Marashdeh M. (2012). Patients' perception of cross-infection prevention in dentistry in Jordan. *Oral Health and Preventive Dentistry*.

[26] Elkarim I. A., Abdulla Z. A., Yahia N. A., AlQudah A., Ibrahim Y. E. (2004). Basic infection control procedures in dental practice in Khartoum—Sudan. *International Dental Journal*.

[27] Rahman B., Abraham S. B., Alsalami A. M., Alkhaja F. E., Najem S. I. (2013). Attitudes and practices of infection control among senior dental students at college of dentistry, University of Sharjah in the United Arab Emirates. *European Journal of Dentistry*.

[28] Bellissimo-Rodrigues W. T., Bellissimo-Rodrigues F., Machado A. A. (2009). Infection control practices among a cohort of Brazilian dentists. *International Dental Journal*.

[29] Acosta-Gío A. E., Borges-Yáñez S. A., Flores M. (2008). Infection control attitudes and perceptions among dental students in Latin America: implications for dental education. *International Dental Journal*.

[30] Singh B. P., Khan S. A., Agrawal N., Siddharth R., Kumar L. (2012). Current biomedical waste management practices and cross-infection control procedures of dentists in India. *International Dental Journal*.

[31] Yüzbasioglu E., Saraç D., Canbaz S., Saraç Y. S., Cengiz S. (2009). A survey of cross-infection control procedures: knowledge and attitudes of Turkish dentists. *Journal of Applied Oral Science*.

[32] Al-Omari M. A., Al-Dwairi Z. N. (2005). Compliance with infection control programs in private dental clinics in Jordan. *Journal of Dental Education*.

[33] AL Negrish A., Al Momani A. S., AL Sharafat F. (2008). Compliance of Jordanian dentists with infection control strategies. *International Dental Journal*.

[34] Al-Rabeah A., Mohamed A. G. (2002). Infection control in the private dental sector in Riyadh. *Annals of Saudi Medicine*.

[35] Chemaitelly H., Chaabna K., Abu-Raddad L. J. (2015). The epidemiology of hepatitis C virus in the fertile crescent: systematic review and meta-analysis. *PLoS ONE*.

[36] Abou Rached A., Abou Kheir S., Saba J., Ammar W. (2016). Epidemiology of hepatitis B and hepatitis C in Lebanon. *Arab Journal of Gastroenterology*.

[37] UNAIDS Global Report: UNAIDS Report on the Global AIDS Epidemic, 2013.

[38] Araj G. F., Saade A., Itani L. Y., Avedissian A. Z. (2016). Tuberculosis burden in Lebanon: evolution and current status. *The Lebanese Medical Journal*.

[39] Ministry of the Environment in Lebanon (MoE) (2004). Specification of the types of healthcare wastes and their disposal methods. *Decree no. 13389*.

[40] Daou M. H., Karam R., Khalil S., Mawla D. (2015). Current status of dental waste management in Lebanon. *Environmental Nanotechnology, Monitoring and Management*.

[41] Ammon A., Reichart P. A., Pauli G., Petersen L. R. (2000). Hepatitis B and C among Berlin dental personnel: incidence, risk factors, and effectiveness of barrier prevention measures. *Epidemiology and Infection*.

[42] El-Sayed A. K., Khalifa G. A. (2015). Dental health care workers at Cairo Dental Research Center: do they need further training on infection control strategy to prevent viral hepatitis transmission. *International Journal of Advanced Research*.

[43] Reddy V., Bennadi D., Kshetrimayum N. (2014). Prevalence of hepatitis B vaccination among oral health care personnel in Mysore city, India.. *Oral Health and Dental Management*.

[44] Shah S. M., Merchant A. T., Dosman J. A. (2006). Percutaneous injuries among dental professionals in Washington State. *BMC Public Health*.

[45] Suljak J. P., Leake J. L., Haas D. A. (1999). The occupational risk to dental anesthesiologists of acquiring 3 bloodborne pathogens.. *Anesthesia Progress*.

[46] Capilouto E. I., Weinstein M. C., Hemenway D., Cotton D. (1992). What is the dentist's occupational risk of becoming infected with hepatitis B or the human immunodeficiency virus?. *American Journal of Public Health*.

[47] Kateeb E., Amer R., Bajali M. (2015). Factors related to the willingness of Palestinian dentists to treat patients with blood-borne diseases. *International Dental Journal*.

[48] Alavian S.-M., Moosavi S.-H., Mousavi S.-H., Azizi B., Akbari H. (2008). Study of admission rate of hepatitis B surface antigen positive patients in 50 dentistry centers in Tehran (Spring 2003). *Hepatitis Monthly*.

[49] El-Maaytah M. A., Jerjes W., Upile T. (2008). Willingness of Jordanian clinicians to treat a hepatitis B-infected patient. *Quintessence International*.

[50] Sadeghi M., Hakimi H. (2009). Iranian dental students' knowledge of and attitudes towards HIV/AIDS patients. *Journal of Dental Education*.

[51] Ellepola A. N. B., Sundaram D. B., Jayathilake S., Joseph B. K., Sharma P. N. (2011). Knowledge and attitudes about HIV/AIDS of dental students from Kuwait and Sri Lanka. *Journal of Dental Education*.

[52] Osazuwa-Peters N. N., Azodo C. C., Ehizele A. O., Obuekwe O. N. (2012). Gender differences in characteristics, occupational exposure, and infection control practices among dental professionals in Edo State, Nigeria. *Southern African Journal of Epidemiology and Infection*.

[53] Al Wazzan K. A., Almas K., Al Qahtani M. Q., Al Shethri S. E., Khan N. (2001). Prevalence of ocular injuries, conjunctivitis and use of eye protection among dental personnel in Riyadh, Saudi Arabia. *International Dental Journal*.

[54] Ghasemi H., Bayat F., Hooshmand B., Maleki Z. (2011). Determinants of Iranian dentists' behaviour regarding infection control. *International Dental Journal*.

[55] Shojaei S., Jamshidi S., Moghimbeigi A., Mostaghimi N. (2011). Evaluation of infection control in dental offices in Hamadan in 2010. *Avicenna Journal of Dental Research*.

[56] Vega O. G., Janus C., Laskin D. M. (2012). Hand-washing knowledge and practices among dentists and dental specialists.. *Quintessence international*.

